# Migrating Brain Cells Stick Together

**DOI:** 10.1371/journal.pbio.1000239

**Published:** 2009-11-10

**Authors:** Mason Inman

**Affiliations:** Freelance Science Writer, Karachi, Pakistan

Slime molds provide a textbook example of self-organization. They live as single cells until food becomes scarce. Then, they broadcast chemical signals that trigger their mass assembly into a fruiting body, with some cells forming a stalk and others turning into spores that cast about in the winds to spread far and wide.

Neurons in the developing brain complete their own self-organized waltz, coordinating with their neighbors to migrate to the right spots to form the cerebellum, visual cortex, or other parts of the brain. In this issue of *PLoS Biology*, Reinhard Köster and colleagues show that some of these brain cells behave much like slime molds, coordinating with other cells of the same type to migrate in a herd. They found that one particular protein called Cadherin-2 is crucial in allowing the cells to adhere to their neighbors so they can coordinate their movements and all wind up in the right spot.

Researchers have long known that proteins known as cadherins—short for calcium-dependent adhesion molecules—play a key role in helping neurons navigate through the developing brain. Cadherins sit within the cell membrane, projecting a chemical hook outside the cell that allows it to adhere to its neighbor. Cells that fail to make these connections fail to differentiate normally and also lose their ability to migrate normally, earlier studies have shown. But the mechanisms behind this coordinated movement—in particular, how each cell adjusts its inner workings to move to the right place at the right time—are only now starting to be revealed, using imaging that tracks these cells in live animals as they develop.

In this new study, Köster and colleagues reveal crucial pieces of this puzzle, showing how the cells orient themselves to migrate together. The team studied zebrafish, one of the workhorses of developmental neurobiology, because its transparent body allows researchers to track movements of cells inside of it.

They focused on one particular cell type called cerebellar granular cells, which form in a region called the cerebellar rhombic lip and then migrate to another zone known as the midbrain–hindbrain boundary, where the cells form the interneuron layer of the cerebellum, the largest neuron layer of the entire brain. To reach their destination, the researchers found that these cells line up and form chains that move forward in steps, with a rest between each step—a microscopic conga line.

To figure out what triggers the cells to line up and move together, the authors looked at what other kinds of cells were in the neighborhood. Many studies have shown that support cells, known as glial cells, often help guide neurons during these kinds of migrations. But during the first few days of the zebrafish embryo's development, Köster and colleagues found, there were no glial cells along the granular cells' migration route. That means these cells must go it alone, the team reasoned, with their own mechanism for signaling between each other to line up into chains and make their move.

Earlier work showed that mutant animals that do not express working copies of the Cadherin-2 protein have serious defects in their cerebellums. Köster and colleagues dug further into the mechanism behind this, showing that mutant granular cells lacking Cadherin-2 migrated in random directions. This seemed to be because the cells were no longer able to coordinate with each other to help them head in the right direction, the new study argues. In some of these mutant zebrafish, the authors gave the animals the ability to temporarily make Cadherin-2, which restored their cerebellums' ability to initially develop normally. This helped them pin down the stage at which Cadherin-2 plays its crucial role in granule cells, showing that although it's not needed for the cells to form in the rhombic lip, it's necessary for them to coordinate migration with their neighbors and follow the normal route.

By developing a Cadherin-2 reporter labeled with a fluorescent dye and capturing it in time-lapse movies, the researchers found that the protein gathered at the front edge of the granular cells each time they were going to make a step forward. Besides adhering to other cells, Cadherin-2 is known to play another role, which could explain why it gathers at the cell's leading edge. On the inner side of the cell membrane, Cadherin-2 connects to a protein complex that, in turn, connects to microtubules—long, tube-shaped molecules that are a key part of the cell's cytoskeleton.

The findings suggest that when Cadherin-2 adheres to a cell's neighbors, this helps organize the microtubules on the leading edge of the cell. This also explains something that had been observed in these migrating brain cells before: Each cell's centrosome, an organelle that is connected to the microtubules and helps organize them, always stays in front of the cell's nucleus, leading the way as the cell migrates. Therefore, concentrating Cadherin-2 at the cell's front should stabilize the centrosome to face in the direction of the next migratory step.

Although the study focused on just one type of brain cell, the findings could explain how many types of neurons find their way to their proper spots as the brain develops. There are still some pieces of the puzzle missing, however. While the findings explain how the granule cells are able to coordinate and follow their neighbors, it's still not clear how the first few cells to head out on the journey—those at the front of the “conga line”—get oriented in the right direction. This suggests there must be some kind of signal from surrounding cells to get them headed in the right direction, the authors argue—yet another level of organization.

**Rieger S, Senghaas N, Walch A, Köster RW (2009) Cadherin-2 Controls Directional Chain Migration of Cerebellar Granule Neurons. doi:10.1371.journal.pbio.1000240**

**Figure:**
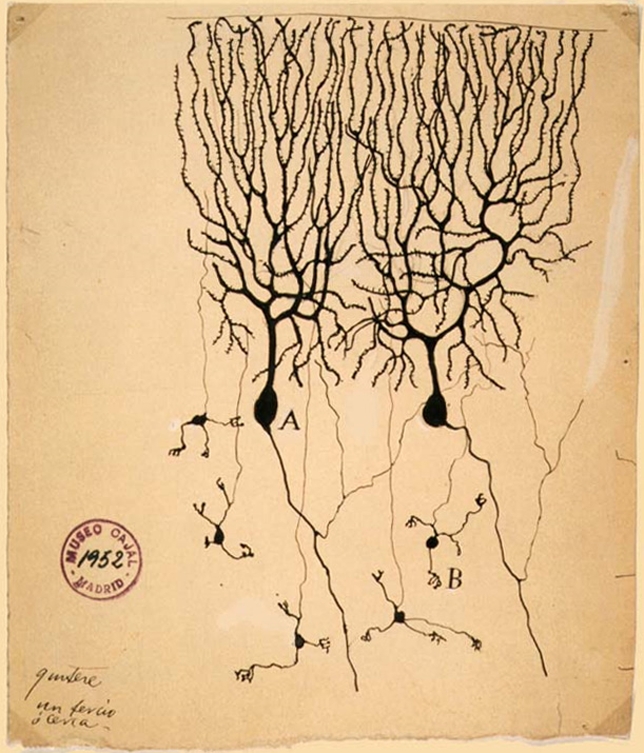
Time-lapse imaging in live zebrafish embryos reveals that cerebellar granule cells migrate in chain-like structures. Granule cells taken from the cerebellum of a pigeon (above, B) are shown in this 1899 drawing by legendary neuroscientist Santiago Ramón y Cajal.

